# Role of peritumoral tissue analysis in predicting characteristics of hepatocellular carcinoma using ultrasound-based radiomics

**DOI:** 10.1038/s41598-024-62457-6

**Published:** 2024-05-21

**Authors:** Hongwei Qian, Yanhua Huang, Luohang Xu, Hong Fu, Baochun Lu

**Affiliations:** 1 Department of Hepatobiliary and Pancreatic Surgery, Shaoxing People’s Hospital, 568 Zhongxing North Road, Shaoxing, 312000 People’s Republic of China; 2 Shaoxing Key Laboratory of Minimally Invasive Abdominal Surgery and Precise Treatment of Tumor, Shaoxing, People’s Republic of China; 3 Department of Ultrasound, Shaoxing People’s Hospital, Shaoxing, People’s Republic of China; 4 School of Medicine, Shaoxing University, Shaoxing, Zhejiang People’s Republic of China

**Keywords:** Cancer imaging, Liver cancer

## Abstract

Predicting the biological characteristics of hepatocellular carcinoma (HCC) is essential for personalized treatment. This study explored the role of ultrasound-based radiomics of peritumoral tissues for predicting HCC features, focusing on differentiation, cytokeratin 7 (CK7) and Ki67 expression, and p53 mutation status. A cohort of 153 patients with HCC underwent ultrasound examinations and radiomics features were extracted from peritumoral tissues. Subgroups were formed based on HCC characteristics. Predictive modeling was carried out using the XGBOOST algorithm in the differentiation subgroup, logistic regression in the CK7 and Ki67 expression subgroups, and support vector machine learning in the p53 mutation status subgroups. The predictive models demonstrated robust performance, with areas under the curves of 0.815 (0.683–0.948) in the differentiation subgroup, 0.922 (0.785–1) in the CK7 subgroup, 0.762 (0.618–0.906) in the Ki67 subgroup, and 0.849 (0.667–1) in the p53 mutation status subgroup. Confusion matrices and waterfall plots highlighted the good performance of the models. Comprehensive evaluation was carried out using SHapley Additive exPlanations plots, which revealed notable contributions from wavelet filter features. This study highlights the potential of ultrasound-based radiomics, specifically the importance of peritumoral tissue analysis, for predicting HCC characteristics. The results warrant further validation of peritumoral tissue radiomics in larger, multicenter studies.

## Introduction

Hepatocellular carcinoma (HCC) continues to pose a substantial global health burden and is ranked as a leading cause of cancer-related mortality worldwide^1^. Despite advancements in diagnostic and therapeutic strategies, the prognosis of liver cancer remains largely contingent on the disease stage and histopathological characteristics. Early and accurate identification of the relevant features is thus crucial for refining treatment approaches and enhancing patient outcomes^2^.

Medical imaging has recently undergone remarkable technological advances, enabling the extraction of intricate quantitative data from radiological images. This evolution has given rise to the field of radiomics^3^, which involves high-throughput extraction, analysis, and interpretation of numerous quantitative image features encapsulating subtle patterns, textures, and spatial relationships that transcend conventional visual assessment. Radiomics demonstrates substantial potential for non-invasive prognostication, treatment response assessment, and early detection across various cancer types^4–6^. Previous studies have demonstrated correlations of differentiation, cytokeratin 7 (CK7), Ki67, and p53 with the invasiveness and prognosis of HCC^7–10^. Utilizing radiomics technology to predict these features preoperatively in patients with HCC would aid the provision of more personalized treatment.

Ultrasound image-based radiomics, as a subset of radiomics, plays a pivotal role in liver cancer research. This method combines medical imaging and computational science to extract quantitative features from ultrasound images, revealing subtle patterns and spatial relationships that are not easily visible to the human eye^9,11^. Analyzing the wealth of information contained within ultrasound images has the potential to transform clinical decision-making and improve patient care in HCC research^12^.

Peritumoral tissue, comprising the area surrounding the tumor, is vitally important in terms of liver cancer research. This domain, including adjacent normal tissue as well as regions potentially influenced by the tumor, plays a pivotal role in tumor development, invasion, treatment response, and prognosis^13,14^. The intricate interplay between tumor cells and the peritumoral microenvironment underscores its relevance in understanding the mechanisms underpinning liver cancer progression. Analyzing the cellular and molecular changes within peritumoral tissues thus offers critical insights into tumor-host interactions, therapeutic effectiveness, and patient outcomes^15^.

The current study aimed to investigate the potential of ultrasound radiomics based on peritumoral tissues to predict the intricate pathological features of HCC. By analyzing concealed information within ultrasound images coupled with advanced computational algorithms, we constructed preoperative models for predicting pathological features in patients with HCC, including differentiation, CK7 and Ki67 expression, and p53 mutation. Integrating ultrasound-based radiomics with peritumoral tissue analysis has the potential for enhancing preoperative assessment accuracy, guiding personalized treatment strategies, and ultimately improving clinical decision-making in liver cancer management.

## Materials and methods

### Study population

This study was carried out in compliance with the Declaration of Helsinki. Written informed consent was obtained from the patients and/or their legal guardian (s). The study was approved by the Ethics Committee of Shaoxing People’s Hospital, and all procedures were carried out in accordance with the relevant guidelines and regulations. We conducted a retrospective analysis of patients with HCC who underwent surgical treatment at our hospital from September 2019 to November 2023. Inclusion and exclusion criteria were established to ensure the selection of appropriate patients. The inclusion criteria were: (1) age ≥ 18 years; (2) pathologically confirmed HCC; (3) ultrasound examination performed within 2 weeks prior to surgery; and (4) patient and family consent to participate in this study. The exclusion criteria were: (1) history of targeted, immunotherapeutic, or other anti-tumor treatments before surgery (n = 7); (2) concurrent other malignancies or a history of malignant tumors (n = 9); (3) suboptimal image quality (n = 5); and (4) incomplete clinical data (n = 29).

The patients were categorized into four subgroups based on distinct pathological features: a differentiation subgroup (n = 130), CK7 subgroup (n = 80), Ki67 subgroup (n = 145), and p53 subgroup (n = 89). Patients in each subgroup were classified as positive or negative (high or low), and then split into a training set and a test set in a 7:3 ratio. Clinical information including age, sex, and serum markers were also collected.

**Figure 1:**
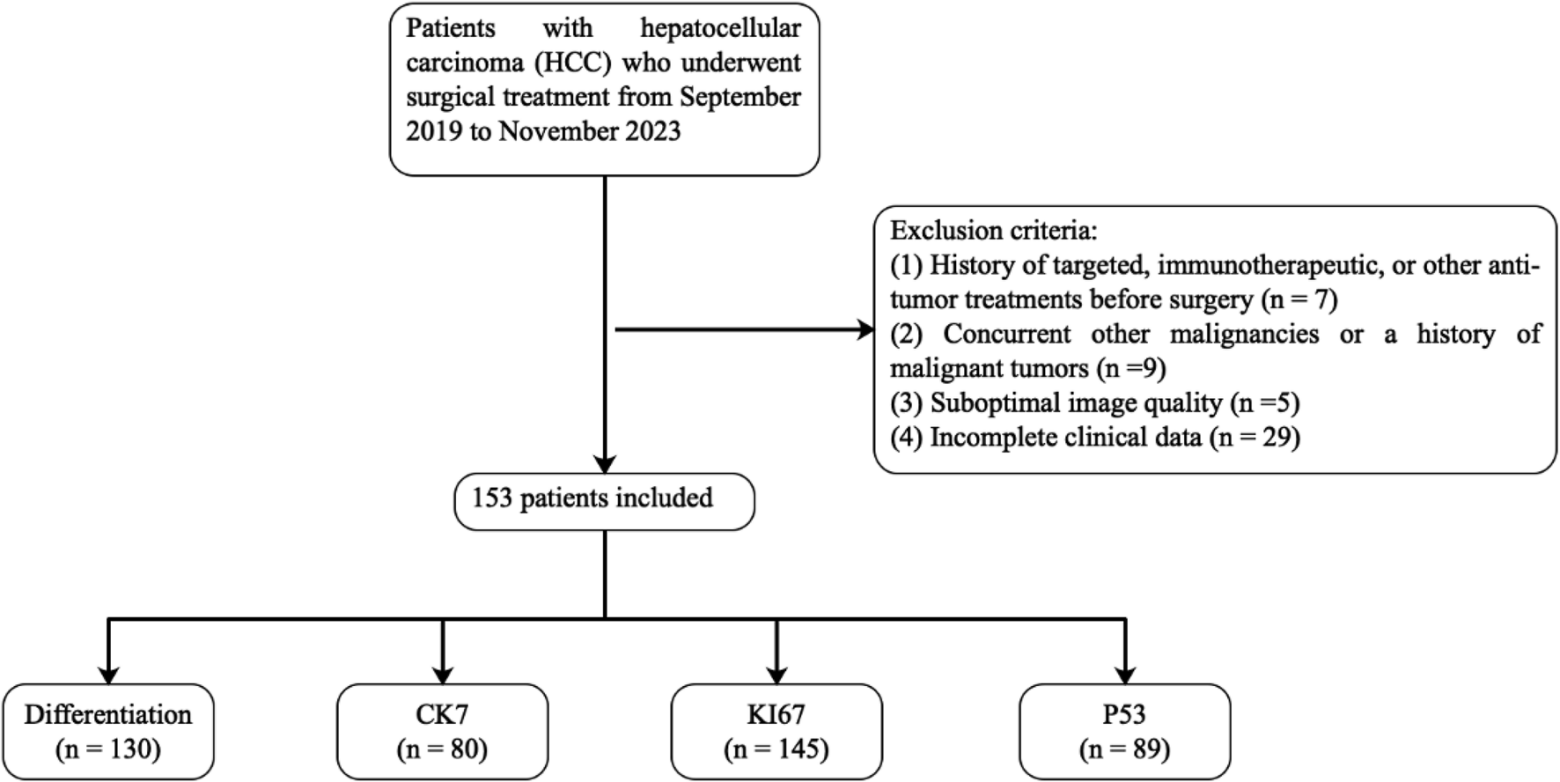
Flowchart of included and excluded patients.

A flowchart illustrating the patient selection process is presented in Fig. 1.

### Ultrasound procedure

All ultrasound examinations were conducted by proficient radiologists following a standardized protocol, to ensure uniformity and accuracy of the imaging data. The patients were scanned in a supine or lateral position with both arms raised, to adequately expose the projection area of the liver region in the field of view. The transducer was placed on the skin surface with an appropriate coupling gel to enhance sound wave transmission and minimize interference. Following routine two-dimensional (2D) ultrasound scans to identify the lesions, the images were adjusted to obtain the optimal display of the lesion. Multiple images were acquired for each patient to capture diverse views of the peritumoral tissues. The resulting images were saved in Digital Imaging and Communications in Medicine (DICOM) format for subsequent analysis. The images containing the largest cross-sections of the tumors were selected for subsequent analysis.

The specific ultrasound machines utilized in this study are detailed in the Supplementary Material.

### Histology and immunohistochemistry

Following surgical resection, tumor specimens were collected and processed for histopathological examination. Tissue sections were prepared from formalin-fixed, paraffin-embedded tumor samples. Hematoxylin and eosin staining was performed to assess tissue morphology and tumor differentiation. Immunohistochemistry was carried out to determine the status of CK7, Ki67, and p53. All pathological assessments of specimens were conducted by experienced pathologists.

### Region of interest delineation

**Figure 2:**
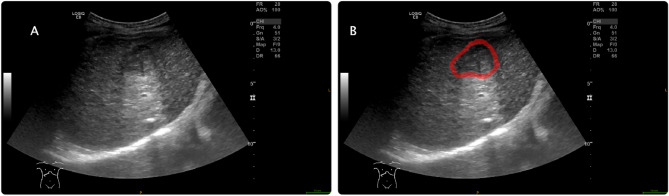
Example of region of interest delineation on ultrasound imaging using ITK-SNAP software. (**A**) The original ultrasound image of the liver lesion. (**B**) The same ultrasound image with ROI outlined in red, demarcating the area around the lesion for subsequent radiomic analysis. ROI, region of interest.

The region of interest (ROI) was delineated independently by two different ultrasound physicians who were unaware of the clinical data, using ITK-SNAP software (Version 3.8.0, https://www.itksnap.org)^16^ (Fig. 2). The two physicians manually outlined the intratumoral ROI along the edges of the lesions. The contour of the surrounding tissues was located 2 cm away from the tumor^9,17^. The peritumoral ROI was initially generated using Python scripts employing the SimpleITK and SciPy packages to automate the contour dilation from the tumor border, with subsequent manual adjustments in ITK-SNAP to refine the delineation accuracy. If the peritumoral area extended beyond the liver tissue boundary, the liver capsule served as the demarcation point. Ultrasound images of the same patient taken 1 week later were again used for ROI delineation to assess inter-observer and intra-observer consistency. The consistency of the ROI drawn twice by the same physician was evaluated by intra-class correlation coefficients, and consistency between the two physicians was assessed by inter-class correlation coefficients.

### Feature extraction and dimension reduction

Before feature extraction, the images underwent a meticulous standardization process to ensure uniformity and consistency across the dataset. This involved several key steps, including resampling the images to achieve a consistent spatial resolution of 3 × 3 × 3 mm^3^, normalizing intensity values to 32 Gy levels using a scale of 255, and effectively eliminating machine-specific artifacts or noise.

Feature extraction was carried out using the PyRadiomics open-source imaging toolkit, encompassing first-order features, 3D and 2D shape features, and texture features. The texture features included gray level co-occurrence matrix features, gray level size zone matrix features, gray level run length matrix features, neighboring gray tone difference matrix features, and gray level dependence matrix features. Subsequent image-filtering methods were applied to the original images for secondary feature extraction. The image-filtering methods comprised Laplacian of Gaussian (based on SimpleITK functionality), wavelet (utilizing the PyWavelets package), square, square root, logarithm, exponential, gradient, local binary pattern (2D), and local binary pattern (3D). Considering the significant disparity in magnitude among different features, Z-score normalization was applied to the extracted feature data to ensure comparability.

Data dimensionality reduction is the first step in constructing radiomics models. This study employed various methods to identify the most stable and relevant features for reducing the data dimension. Inter- and intra-class coefficients (ICC) were calculated and an ICC > 0.8 was considered indicative of good consistency. Spearman’s correlation coefficient was then employed to assess the correlations among features, and features with correlation coefficients > 0.80 were systematically excluded from subsequent analyses to ensure the retention of only minimally correlated features. The final radiomics features incorporated into the model construction were then selected by *t*-tests and the least absolute shrinkage and selection operator (LASSO) method.

### Radiomics model construction

We constructed predictive models using a variety of modeling techniques and enhanced the predictive performance of the models using a combination of RandomizedSearchCV and GridSearchCV to optimize the model parameters. We initially used RandomizedSearchCV to identify the approximate range of optimal parameters for the model, and subsequently applied GridSearchCV within this range to further refine and obtain the best parameters.

After feature dimensionality reduction, we utilized various modeling techniques including support vector machine (SVM), random forest, K nearest neighbor, logistic regression, decision tree, artificial neural network, AdaBoostClassifier, GradientBoostingClassifier, and XGBOOST. We conducted modeling through fivefold cross-validation and performed receiver operating characteristic (ROC) curve analysis, and calculated the corresponding area under the curve (AUC). The model with the highest AUC in the test group was selected as the predictive model, and further validation was carried out.

### Statistical analysis

The radiomics procedures and the statistical analyses were all conducted using Python (Version 3.11). Depending on the normality of their distribution, continuous variables were either presented as mean ± standard deviation or median and range. The significance of continuous variables was evaluated by *t*-tests or Mann–Whitney U tests, depending on the distribution of the information. Categorical variables were evaluated by χ^2^ or Fisher’s tests. A significance threshold of p < 0.05 was adhered to in all analyses.

**Figure 3:**
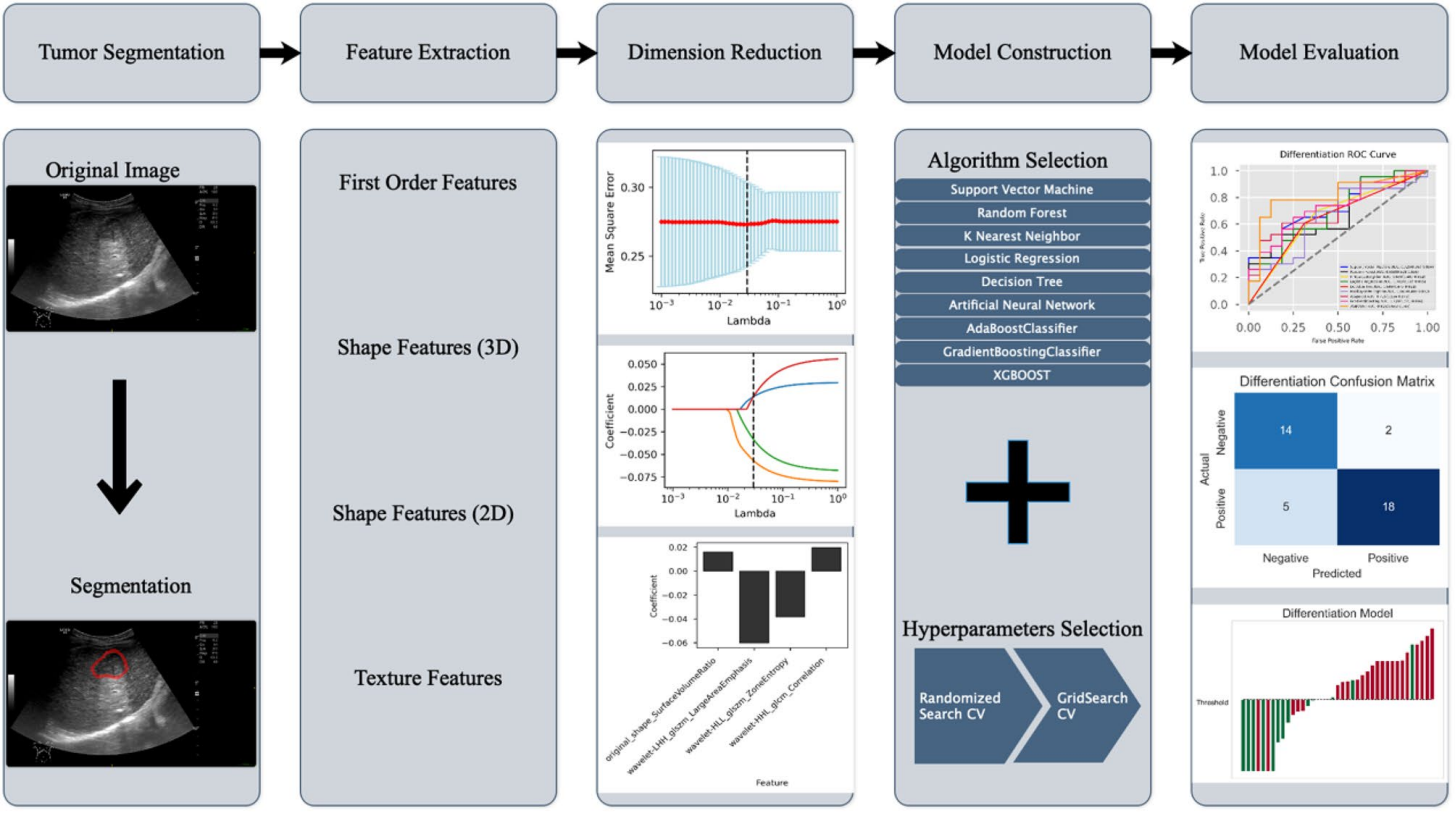
Radiomics workflow showcasing the process from tumor segmentation to model evaluation, as applied to the differentiation model.

The radiomics workflow chart is shown in Fig. 3.

### Ethics approval and consent to participate

The study was approved by the Ethics Committee of Shaoxing People’s Hospital, and all procedures were carried out in accordance with the relevant guidelines and regulations. This study was carried out in compliance with the Declaration of Helsinki. Written informed consent was obtained from the patients and/or their legal guardian (s).

## Results

### Characteristics of the study population

A total of 153 patients (121 males and 32 females) were included in this study and their clinical data are presented in Table 1. Patients within each subgroup were divided randomly into training and test groups at a ratio of 7:3 (detailed clinical information provided in Supplementary Table S1). Subgroup sizes varied because of incomplete datasets for some patients.

**Table 1:** Demographic and clinical characteristics of patients.

Variables	Differentiation (n = 130)			CK7 (n = 80)			KI67 (n = 145)			P53 (n = 89)		
	Low (n = 79)	High (n = 51)	p	Negative (n = 56)	Positive (n = 24)	p	Low (n = 71)	High (n = 74)	p	Negative (n = 62)	Positive (n = 27)	p
Age (year)	63.61 ± 10.84	66.88 ± 9.88	0.087	63.52 ± 10.25	64.67 ± 11.3	0.661	66.48 ± 10.13	62.89 ± 10.96	0.044*	65.0 ± 10.97	62.11 ± 9.54	0.244
AFP (mg/mL)	2243.43 ± 10,239.63	785.16 ± 4797.15	0.347	1704.19 ± 6055.3	51.4 ± 140.19	0.191	830.48 ± 4240.29	2523.21 ± 10,643.21	0.217	1372.26 ± 6206.16	4527.33 ± 15,881.4	0.187
ALT (IU/L)	38.35 ± 34.14	40.78 ± 40.26	0.715	34.82 ± 37.27	40.2 ± 29.94	0.538	38.92 ± 34.56	38.88 ± 36.08	0.995	35.13 ± 31.47	36.39 ± 26.19	0.857
AST (IU/L)	44.43 ± 42.96	43.15 ± 39.45	0.866	43.08 ± 49.15	36.11 ± 16.99	0.505	44.9 ± 34.67	44.56 ± 46.4	0.961	39.67 ± 34.87	38.92 ± 19.43	0.918
TBIL (µmol/L)	17.83 ± 18.7	18.29 ± 10.75	0.874	19.38 ± 21.01	19.08 ± 12.64	0.95	16.9 ± 8.77	18.63 ± 19.75	0.501	15.46 ± 10.57	16.27 ± 11.63	0.752
DBIL (µmol/L)	6.26 ± 10.86	5.73 ± 4.69	0.744	6.58 ± 12.43	5.46 ± 5.8	0.679	5.8 ± 4.7	6.24 ± 11.06	0.757	5.33 ± 4.88	4.6 ± 4.98	0.524
Alb (g/L)	39.38 ± 3.83	38.59 ± 4.29	0.275	39.31 ± 4.58	39.53 ± 3.5	0.837	38.7 ± 4.26	38.69 ± 4.47	0.997	38.82 ± 3.9	38.96 ± 5.5	0.893
PT (s)	12.83 ± 1.25	13.04 ± 1.54	0.39	12.89 ± 1.54	12.51 ± 0.97	0.266	13.01 ± 1.31	12.96 ± 1.42	0.835	13.08 ± 1.5	12.55 ± 1.14	0.106
INR	1.03 ± 0.09	1.06 ± 0.15	0.177	1.05 ± 0.15	1.04 ± 0.08	0.726	1.05 ± 0.11	1.04 ± 0.13	0.563	1.05 ± 0.14	1.05 ± 0.1	0.997
Tumor size (cm)	4.5 (2.5-6.75)	4.0 (2.8-5.05)	0.053	5.09 ± 2.97	4.25 ± 1.98	0.217	4.88 ± 3.02	4.81 ± 2.66	0.886	5.26 ± 2.9	4.73 ± 2.98	0.437
Sex			0.059			0.161			0.377			0.84
Female	22	7		15	3		13	18		15	6	
Male	57	44		41	21		58	56		47	21	
HBsAg			0.666			0.915			0.599			0.376
Negative	22	16		17	7		22	20		22	7	
Positive	57	35		39	17		49	54		40	20	
Cirrhosis			0.98			0.434			0.219			0.014
Absent	37	24		31	11		36	30		36	8	
Present	42	27		25	13		35	44		26	19	
Multifocality			0.341			0.348			0.504			0.377
Absent	63	44		44	21		57	56		51	20	
Present	16	7		12	3		14	18		11	7	

*AFP* alpha fetoprotein, *ALB* albumin level, *ALT* alanine aminotransferase, *AST* aspartate aminotransferase, *TBIL* total bilirubin, *DBIL* directed bilirubin, *PT* prothrombin time, *INR* international normalized ratio.

*p < 0.05.

There was a significant difference in age between the high-Ki67 and low-Ki67 expression groups (66.48 ± 10.13 vs. 62.89 ± 10.96 years, p = 0.044), but no significant disparities in age within the corresponding training and test groups (p > 0.05). Furthermore, there were no significant differences in any clinical parameters across the overall dataset, training group, or test group.

### Feature selection

We extracted a total of 1414 radiomics features from both the original and filtered ultrasound images. After intra- and inter-group analyses, all features demonstrated inter-class correlation coefficients > 0.80, and 1379 of 1414 features had intra-class correlation coefficients above this threshold. Features with high Spearman’s correlation coefficients (> 0.75) were subsequently excluded to minimize redundancy. Further refinements by *t*-tests and LASSO regression resulted in four radiomics features in the differentiation group, three radiomics features in the CK7 group, two radiomics features in the Ki67 group, and six radiomics features in the p53 group (Table 2).

**Table 2:** Final selected features and coefficient values.

Group	Filter	Feature class	Feature	Coefficient
Differentiation	original	shape	SurfaceVolumeRatio	0.015969901
	wavelet-LHH	glszm	LargeAreaEmphasis	-0.06009957
	wavelet-HLL	glszm	ZoneEntropy	-0.038229075
	wavelet-HHL	glcm	Correlation	0.019739638
CK7	wavelet-LHL	firstorder	Skewness	0.11451479
	wavelet-HLH	glszm	GrayLevelNonUniformityNormalized	0.041666225
	logarithm	glszm	SmallAreaLowGrayLevelEmphasis	-0.109409704
KI67	wavelet-LLL	glszm	SmallAreaHighGrayLevelEmphasis	0.062018693
	logarithm	glrlm	ShortRunLowGrayLevelEmphasis	-0.064031164
P53	original	ngtdm	Busyness	-0.061904644
	wavelet-LHH	glszm	SizeZoneNonUniformityNormalized	0.171238122
	wavelet-HLL	firstorder	Skewness	0.114558781
	wavelet-HHH	glszm	HighGrayLevelZoneEmphasis	0.149226288
	wavelet-LLL	gldm	DependenceNonUniformityNormalized	0.024752477
	wavelet-LLL	gldm	DependenceVariance	-0.002089695

### Model construction

We employed a diverse array of modeling techniques, including SVM, random forest, K nearest neighbor, logistic regression, decision tree, artificial neural network, AdaBoostClassifier, GradientBoostingClassifier, and XGBOOST.

A two-stage approach involving RandomizedSearchCV and GridSearchCV was utilized to select the best hyperparameters. The detailed results are shown in the Supplementary Material.

**Figure 4:**
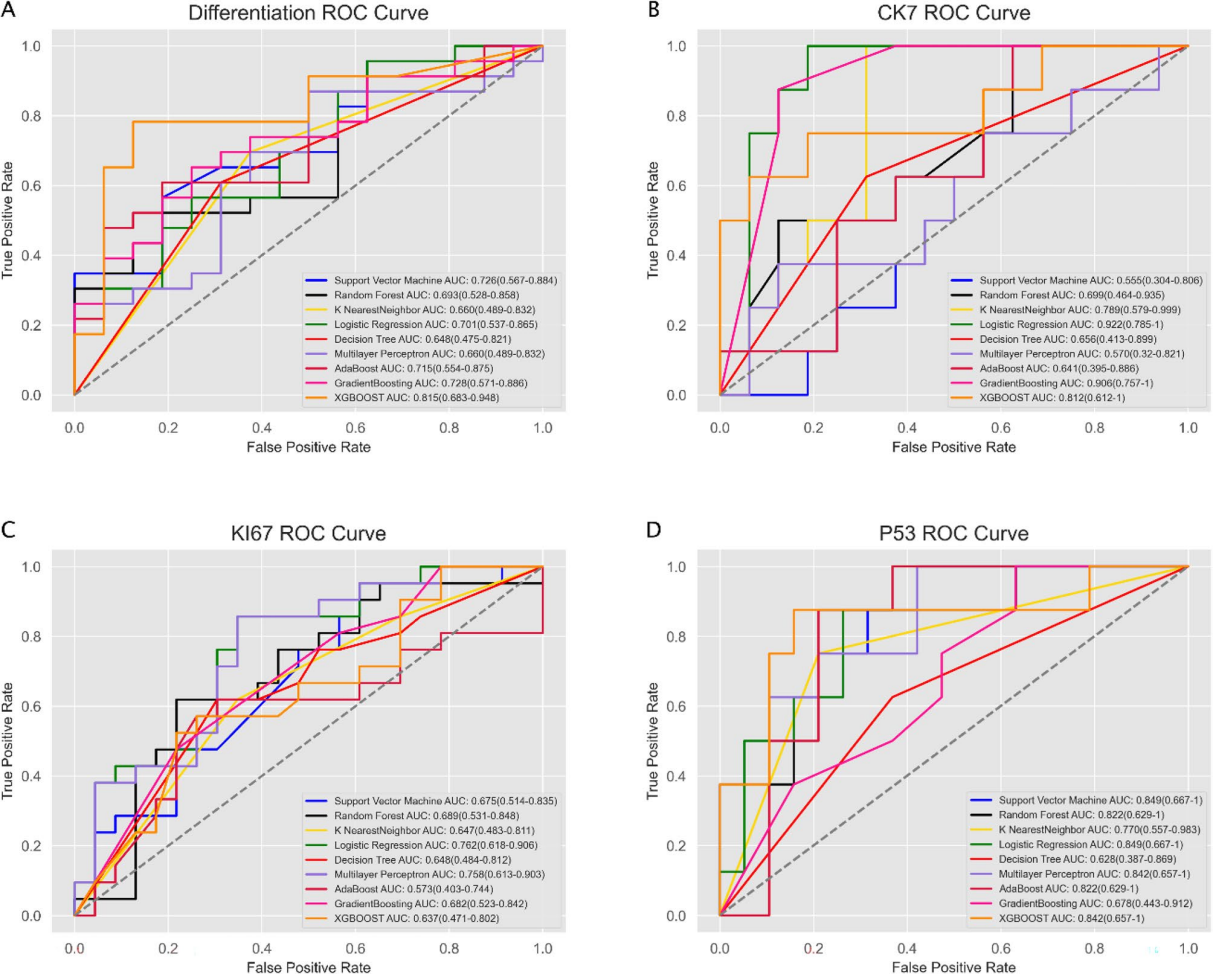
Receiver operating characteristic curve (ROC) analysis of modeling methods in four groups. The XGBOOST algorithm exhibited superior diagnostic performance with an AUC of 0.815 (0.683–0.948) in the differentiation group (**A**). The logistic regression algorithm demonstrated the most effective diagnostic performance in the CK7 group [AUC 0.922 (0.785–1)] (**B**) and Ki67 group [AUC 0.762 (0.618–0.906)] (**C**). The support vector machine algorithm presented the highest diagnostic performance with an AUC of 0.849 (0.667–1) in the p53 group (**D**).

In the differentiation subgroup, the XGBOOST algorithm achieved the highest AUC of 0.815 (0.683–0.948), while the logistic regression algorithm attained the maximum AUCs of 0.922 (0.785–1) in the CK7 subgroup and 0.762 (0.618–0.906) in the Ki67 subgroup, and the SVM algorithm obtained the maximum AUC of 0.849 (0.667–1) in the p53 subgroup. ROC curves for the four subgroups are illustrated in Fig. 4. The detailed performance metrics of the models are presented in Table 3.

**Table 3:** Performance of the differentiation, CK7, Ki67, and p53 models.

	Differentiation	CK7	KI67	P53
TP	18	8	18	8
FP	2	3	8	8
FN	5	0	3	0
TN	14	13	15	11
AUC	0.815217391	0.921875	0.761904762	0.848684211
TPR	0.782608696	1	0.857142857	1
TNR	0.875	0.8125	0.652173913	0.578947368
Precision	0.9	0.727272727	0.692307692	0.5
Recall	0.782608696	1	0.857142857	1
Accuracy	0.820512821	0.875	0.75	0.703703704
F1 score	0.837209302	0.842105263	0.765957447	0.666666667

*TP* true positive, *FP* false positive, *FN* false negative, *TN* true negative, *AUC* area under the curve, *TPR* true positive rate, *TNR* true negative rate.

**Figure 5:**
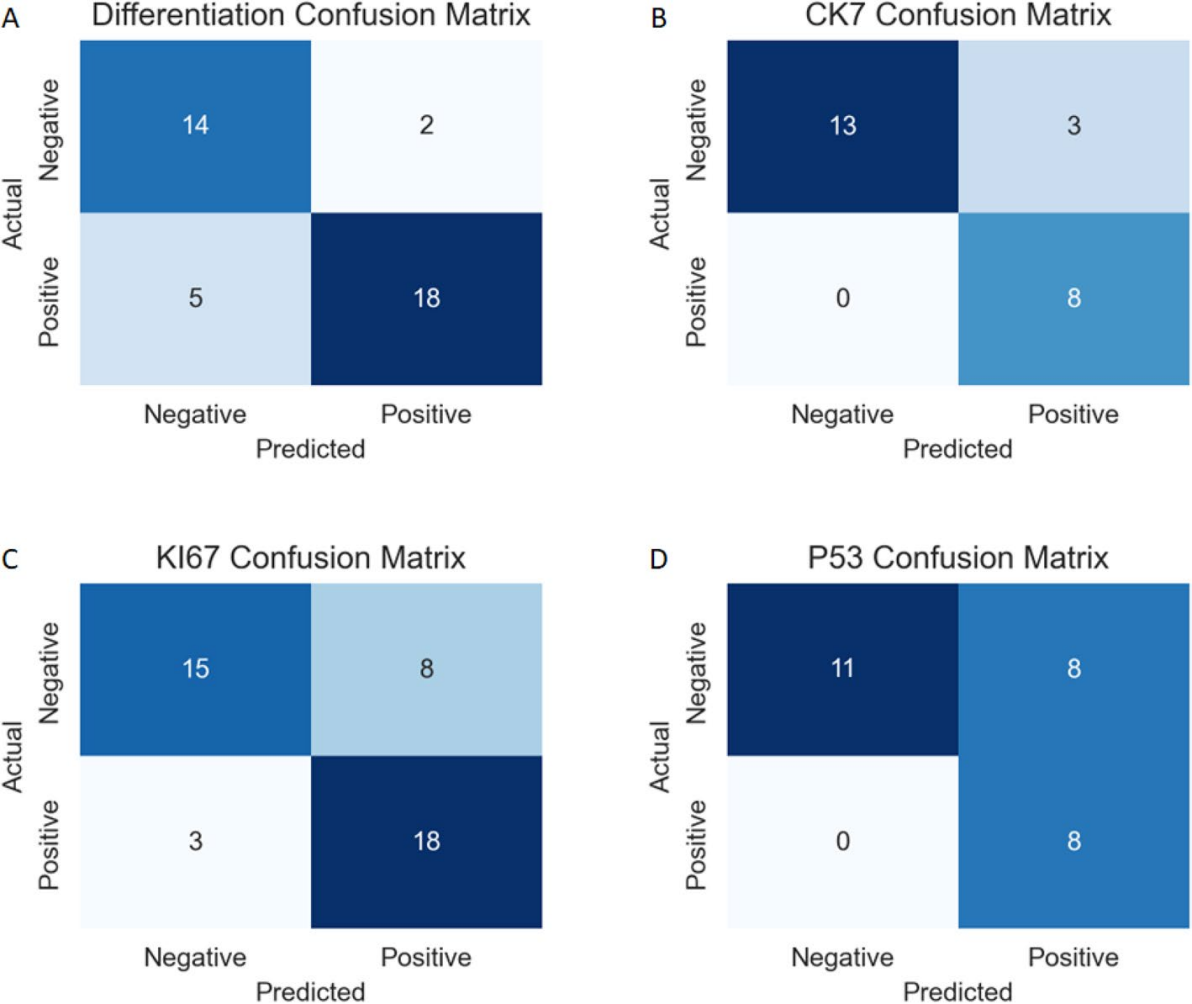
Confusion matrices of the predictive model constructed using the best modeling method in each test set. (**A**) Differentiation group, (**B**) CK7 group, (**C**) Ki67 group, (**D**) p53 group. Low differentiation and high Ki67 expression were defined as positive and high differentiation and low Ki67 expression were defined as negative.

**Figure 6:**
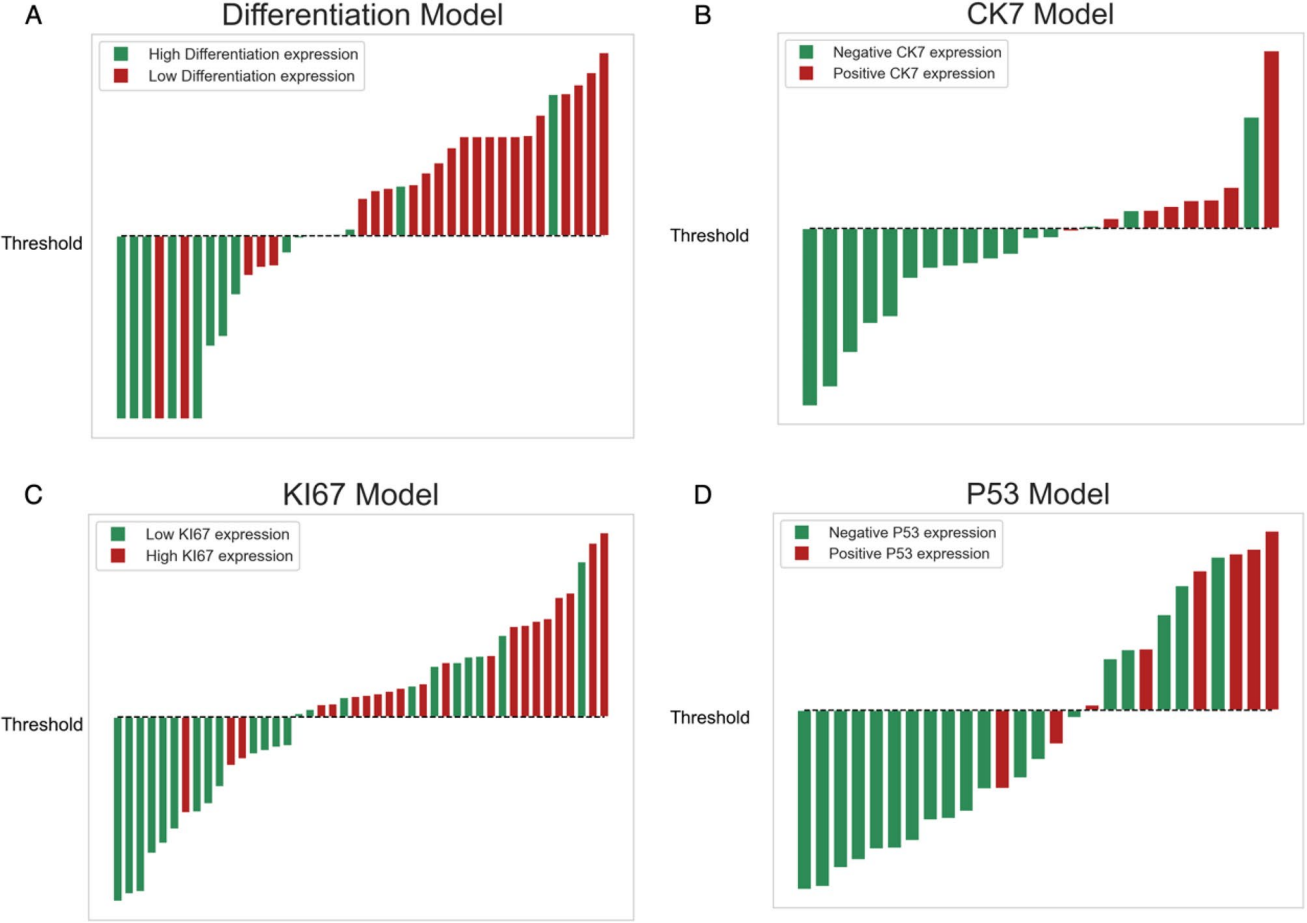
Waterfall plot displaying model performance in the differentiation group (**A**), CK7 group (**B**), Ki67 group (**C**), and p53 group (**D**). Bars above the threshold line indicate a positive prediction and bars below the threshold indicate a negative prediction. Red bars represent actual positive-status cases and green bars represent actual negative-status cases.

To provide a comprehensive assessment of model performance, we included confusion matrices for each model (Fig. 5). Low differentiation and high Ki67 expression were defined as positive and high differentiation and low Ki67 expression were defined as negative. In the waterfall plots, bars above the threshold line indicated a positive prediction and bars below the threshold indicated a negative prediction. Additionally, red bars represent actual positive-status cases and green bars represent actual negative-status cases (Fig. 6).

**Figure 7:**
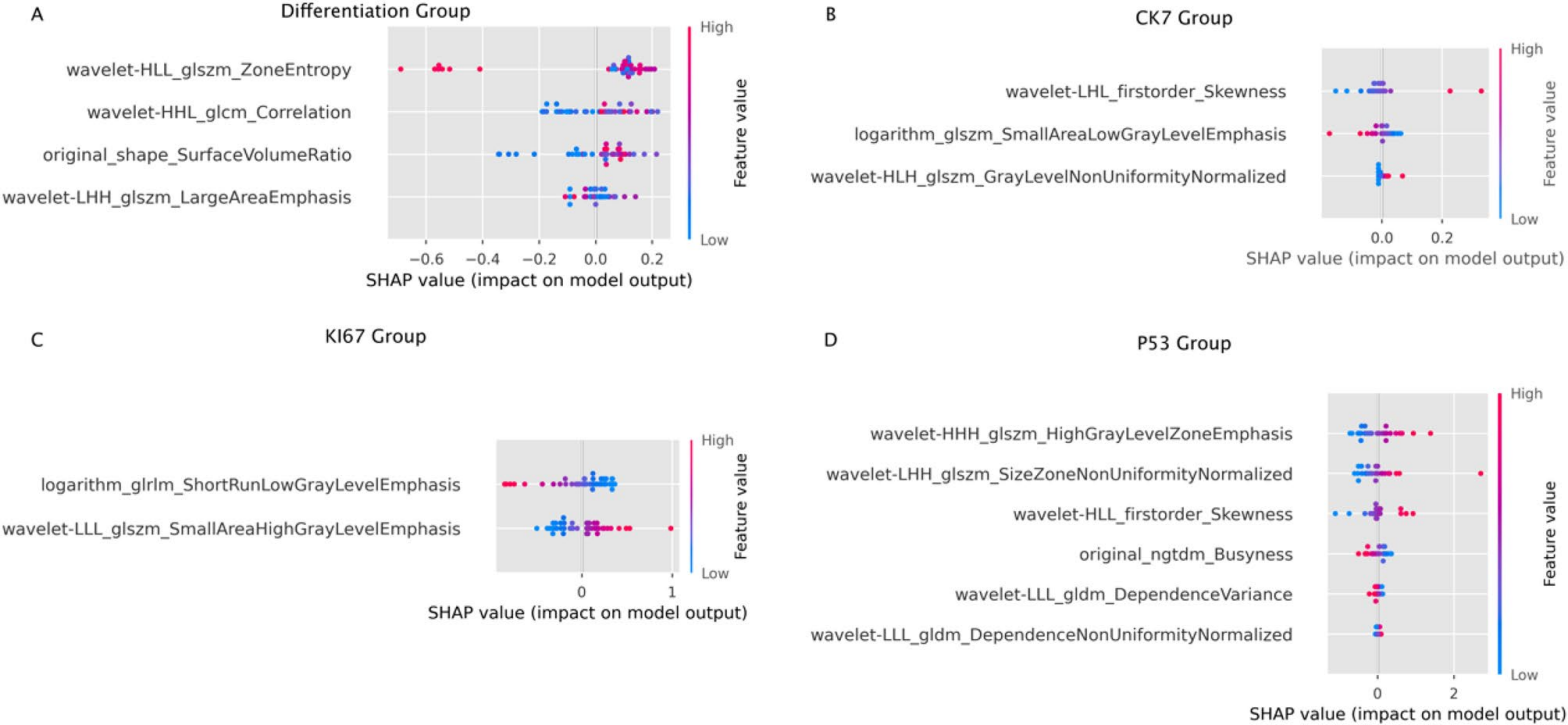
SHAP plots illustrating the impact and contribution of each radiomics feature to the model in the differentiation group (**A**), CK7 group (**B**), Ki67 group (**C**), and p53 group (**D**).

SHapley Additive exPlanations (SHAP) plots are a tool for interpreting the predictions of machine learning models based on the Shapley value principle from cooperative game theory. SHAP plots are used to reveal the contribution of each feature to the model output, enhancing interpretability of machine learning models. To clarify the roles of the different radiomics features in the models, SHAP plots were generated for each model and the results indicated that the wavelet filter had the greatest contribution in most models (Fig. 7).

## Discussion

In this study, we analyzed ultrasound-based radiomics features of peritumoral tissues to predict various biological characteristics of HCC. To the best of our knowledge, this is the first comprehensive study to evaluate the relationship between ultrasound-based radiomics features of peritumoral tissues and the biological properties of HCC. The results demonstrate that ultrasound-based radiomics focusing on peritumoral tissues can accurately predict the biological characteristics of HCC. This provides a novel perspective compared with traditional intratumoral radiomics approaches, which have been the main focus of previous radiomics studies of HCC^18^, suggesting that radiomics analysis of peritumoral tissues may prove equally crucial in future studies.

Constructing predictive models for the biological characteristics of HCC using radiomics technology has recently emerged as a research focus, with noteworthy success^19,20^; however, prior radiomics studies were predominantly centered on the tumor itself, with limited exploration of peritumoral tissues. In addition, such studies often considered peritumoral tissues as supplementary analyses to tumor-focused investigations, resulting in a lack of in-depth radiomics analyses focused exclusively on peritumoral tissues^17,21^. Nevertheless, peritumoral tissues play a pivotal role in the development and invasion of liver cancer^22^. The exploration of radiomics analysis of peritumoral tissues and the development of predictive models thus represent an intriguing and promising avenue of research.

In the current study, we successfully constructed four models for the biological characteristics of HCC, encompassing the important indicators differentiation, CK7, Ki67, and p53. Each of these indicators has profound biological significance, providing crucial insights into the comprehensive understanding of the molecular characteristics of HCC. The differentiation indicator reflects the degree of differentiation of tumor cells. Accurate prediction of this indicator contributes to an understanding of the differentiation status of liver cancer cells, thus providing robust support for the design of treatment strategies. Compared with highly differentiated tumors, poorly differentiated tumors typically exhibit faster growth rates and relatively poorer treatment responses^23^. The cytokeratin CK7 is an important immunohistochemical marker in liver cancer research. The expression pattern of CK7 helps to determine the origin of the tumor cells, determine the likelihood of intrahepatic lymph node metastasis, and assess the prognosis of patients with HCC^24^. Expression of the proliferation marker Ki67 correlates directly with the proliferative activity of tumor cells^25^. Elevated expression of Ki67 may imply a more invasive tumor, thus allowing for a more accurate prediction of patient prognosis and treatment response. Finally, the tumor suppressor protein p53 plays a critical role in various cancers, and influences the development, immune response, and treatment outcomes of liver cancer^26,27^. The integration of these indicators forms a comprehensive predictive model, offering in-depth insights into the molecular biology of liver cancer. This not only improves the accuracy of distinguishing between subtypes of liver cancer, but also provides a reliable foundation for the development of personalized treatment strategies.

The peritumoral area is more than just a transitional zone between tumor tissues and normal liver tissues; it also significantly impacts the tumor’s growth, invasion, metastasis, and resistance to treatment^14,28^. Analyzing peritumoral tissues using radiomics technology is a promising research direction, and despite limited research in this field, some studies have achieved notable success. Yu et al.^29^ developed a radiomics model based on Gd-EOB-DTPA-enhanced magnetic resonance imaging for preoperative prediction of vesicle-encapsulated tumor clusters and patient prognosis in patients with HCC, and showed that both intratumoral and peritumoral radiomics models could effectively predict vesicle-encapsulated tumor clusters and patient prognosis preoperatively. Notably, radiomics models focused on the peritumoral region might have higher predictive value than intratumoral models. In the current study, radiomics models based on peritumoral tissues also demonstrated favorable diagnostic performance, particularly in predicting CK7 expression, with an AUC of 0.922. These findings robustly demonstrate the significant potential of radiomics models based on peritumoral tissues for predicting the biological characteristics of HCC, particularly the accurate prediction of CK7 expression.

We analyzed the impacts of different radiomics features on the four models and found that wavelet filter features had the highest contribution to the model within the differentiation, CK7, and p53 subgroups. This finding highlights the essential role of wavelet analysis in predicting pathological outcomes, in accord with previous research findings^30^. Wavelet filters, commonly employed in image processing, segment images into distinct frequency components, allowing the analysis of image details across various frequencies. This approach facilitates the exploration of spatial heterogeneity within the ROI at multiple scales. The heightened contribution of wavelet features in radiomics suggests the crucial role of frequency domain information from peritumoral tissues in predicting model outcomes within these subgroups ^31^. This may indicate a significant correlation between specific frequency domain features in peritumoral tissues and the differentiation degree, CK7 expression, and p53 mutation status in HCC. These findings acknowledge the use of wavelet features for predicting pathological characteristics in HCC, underscoring the potential of multiscale analysis in radiomics research. This also highlights the critical importance of the meticulous analysis of various features to elucidate their roles in the diagnosis and prediction of liver cancer.

The current study found no significant differences across various clinical parameters. This consistency might be influenced by the characteristics of the study population and could reflect the limited utility of these clinical parameters for predicting specific biological features of HCC^32^. Despite the absence of significant differences however, we emphasize the importance of conducting more in-depth analyses of these clinical parameters in future research, to gain a comprehensive understanding of their potential implications for the development and treatment response in HCC.

This study had several limitations that need to be addressed in future research. First, it was a single-center study with a relatively small sample size, particularly in the CK7 and p53 subgroups, which had sample sizes < 90. Further larger-scale, multicenter studies are therefore required to validate and generalize our findings. Second, the ultrasound data were obtained from different ultrasound devices and the potential impact of equipment-related variations cannot be completely ruled out. Although we standardized the images, there may still have been some residual effects on the model. Third, because grayscale ultrasound is a 2D imaging modality, we only included the plane with the largest tumor cross-section in the study, which may have resulted in the loss of tumor information compared with 3D imaging. Fourth, the inherent variability in ultrasound presets and body positioning during imaging could introduce inconsistencies in image quality and interpretation, although such variability may be seen as an aspect of the adaptability and robustness of our models in real-world clinical settings. Finally, although our models based on peritumoral radiomics features demonstrated promising diagnostic efficacy, we did not conduct a direct comparison with intratumoral-based radiomics models. The inclusion of intratumoral analysis could offer a comprehensive understanding of the radiomics landscape in HCC and potentially validate the superiority of peritumoral analysis in certain contexts.

## Conclusion

We constructed multiple predictive models based on peritumoral ultrasound radiomics to forecast various biological indicators of HCC, with excellent diagnostic performance. The findings indicate a correlation between peritumoral tissues and certain biological characteristics of HCC. It is therefore essential to include peritumoral tissues in future radiomics research of HCC.

### Supplementary Information

Supplementary Information 1.

Supplementary Table S1.

## Data Availability

All data generated or analyzed during this study are included in this published article. Further inquiries can be directed to the corresponding author on reasonable request.

## Abbreviations

HCC: Hepatocellular carcinoma
DICOM: Digital imaging and communications in medicine
ROI: Region of interest
ICC: Inter-/intra-class coefficients
LASSO: Least absolute shrinkage and selection operator
ROC: Receiver operating characteristic
AUC: Area under the curve
SHAP: SHapley Additive exPlanations
SVM: Support vector machine
