# Supplementary material for: Role of peritumoral tissue analysis in predicting characteristics of hepatocellular carcinoma using ultrasound-based radiomics
Source: Sci Rep. 2024 May 21;14:11538. doi: 10.1038/s41598-024-62457-6 (PMC11109225; doi:10.1038/s41598-024-62457-6)
Supplement: Supplementary file 1 — Supplementary Information 1. [file 41598_2024_62457_MOESM1_ESM.docx]

1. **Ultrasound machines**

Ultrasound examination was performed by using one of the following ultrasound machines: LOGIQ E8 (GE Healthcare, United States; C5-1 convex array probes, 1–5 MHz); LOGIQ E9 (GE Healthcare, United States; C5-1 convex array probes, 1–5 MHz); Aplio 500 (Toshiba Medical systems, Japan; 6C1 probe, 1–6 MHz); i800 (Cannon Medical systems Corporation, Japan; i8CX1 probe, 1-8MHz); and Resona 7T (Mindray, China; SC6-1 U probe, 1-6MHz).

1. **Best parameters in Differentiation group**

| **Model** | **Best GridSearch Parameters** |
| --- | --- |
| SVM | {'C': 2.009233002565046, 'gamma': 1244.328681893538, 'kernel': 'sigmoid'} |
| RandomForest | {'max_depth': 10, 'min_samples_split': 5, 'n_estimators': 50} |
| KNN | {'n_neighbors': 1, 'p': 1, 'weights': 'uniform'} |
| LogisticRegression | {'C': 0.04, 'penalty': 'l2'} |
| DecisionTree | {'max_depth': 20, 'min_samples_split': 2} |
| MLPClassifier | {'activation': 'relu', 'alpha': 7.727272727272726e-06, 'hidden_layer_sizes': (50, 50), 'learning_rate': 'invscaling'} |
| AdaBoostClassifier | {'learning_rate': 0.1, 'n_estimators': 250} |
| GradientBoostingClassifier | {'learning_rate': 1.0, 'max_depth': 5, 'n_estimators': 100} |
| XGBOOST | {'gamma': 0.01, 'learning_rate': 0.01, 'max_depth': 5, 'n_estimators': 50} |

1. **Best parameters in CK7 group**

| **Model** | **Best GridSearch Parameters** |
| --- | --- |
| SVM | {'C': 0.7863877800034581, 'gamma': 6.470130633631748, 'kernel': 'rbf'} |
| RandomForest | {'max_depth': None, 'min_samples_split': 2, 'n_estimators': 50} |
| KNN | {'n_neighbors': 7, 'p': 1, 'weights': 'distance'} |
| LogisticRegression | {'C': 0.008, 'penalty': 'l2'} |
| DecisionTree | {'max_depth': None, 'min_samples_split': 2} |
| MLPClassifier | {'activation': 'relu', 'alpha': 4.000000000000001e-06, 'hidden_layer_sizes': (50, 50), 'learning_rate': 'constant'} |
| AdaBoostClassifier | {'learning_rate': 1.0, 'n_estimators': 200} |
| GradientBoostingClassifier | {'learning_rate': 100.0, 'max_depth': 7, 'n_estimators': 100} |
| XGBOOST | {'gamma': 0.01, 'learning_rate': 0.01, 'max_depth': 5, 'n_estimators': 50} |

1. **Best parameters in KI67 group**

| **Model** | **Best GridSearch Parameters** |
| --- | --- |
| SVM | {'C': 97.47336550586843, 'gamma': 199.58831984534962, 'kernel': 'sigmoid'} |
| RandomForest | {'max_depth': 20, 'min_samples_split': 10, 'n_estimators': 250} |
| KNN | {'n_neighbors': 2, 'p': 1, 'weights': 'uniform'} |
| LogisticRegression | {'C': 0.2, 'penalty': 'l2'} |
| DecisionTree | {'max_depth': 30, 'min_samples_split': 10} |
| MLPClassifier | {'activation': 'logistic', 'alpha': 6.500000000000001e-05, 'hidden_layer_sizes': (100,), 'learning_rate': 'invscaling'} |
| AdaBoostClassifier | {'learning_rate': 1.0, 'n_estimators': 50} |
| GradientBoostingClassifier | {'learning_rate': 0.001, 'max_depth': 3, 'n_estimators': 100} |
| XGBOOST | {'gamma': 0.01, 'learning_rate': 0.01, 'max_depth': 7, 'n_estimators': 50} |

1. **Best parameters in P53 group**

| **Model** | **Best GridSearch Parameters** |
| --- | --- |
| SVM | {'C': 0.025972567686498357, 'gamma': 3.0538555088334185, 'kernel': 'linear'} |
| RandomForest | {'max_depth': 20, 'min_samples_split': 2, 'n_estimators': 300} |
| KNN | {'n_neighbors': 1, 'p': 1, 'weights': 'uniform'} |
| LogisticRegression | {'C': 0.012, 'penalty': 'l2'} |
| DecisionTree | {'max_depth': 30, 'min_samples_split': 2} |
| MLPClassifier | {'activation': 'relu', 'alpha': 0.034, 'hidden_layer_sizes': (30, 20, 10), 'learning_rate': 'constant'} |
| AdaBoostClassifier | {'learning_rate': 0.1, 'n_estimators': 100} |
| GradientBoostingClassifier | {'learning_rate': 100.0, 'max_depth': 7, 'n_estimators': 50} |
| XGBOOST | {'gamma': 0.1, 'learning_rate': 1.0, 'max_depth': 3, 'n_estimators': 150} |
