# Supplementary material for: Role of peritumoral tissue analysis in predicting characteristics of hepatocellular carcinoma using ultrasound-based radiomics
Source: Sci Rep. 2024 May 21;14:11538. doi: 10.1038/s41598-024-62457-6 (PMC11109225; doi:10.1038/s41598-024-62457-6)
Supplement: Supplementary file 2 — Supplementary Table S1. [file 41598_2024_62457_MOESM2_ESM.docx]

**Table S1**

**Comparison of clinical characteristics between the training group and testing groups**

| **Variables** |  | **Differentiation(n = 130)** | | | | | |  | **CK7(n=80)** | | | | | |
| --- | --- | --- | --- | --- | --- | --- | --- | --- | --- | --- | --- | --- | --- | --- |
|  |  | **Training Group(n=91)** | |  | **Test Group(n=39)** | |  |  | **Training Group(n=56)** | |  | **Test Group(n=24)** | |  |
|  |  | **Low(n=63)** | **High(n=28)** | **p** | **Low(n=16)** | **High(n=23)** | **p** |  | **Negative(n=40)** | **Positive(n=16)** | **p** | **Negative(n=16)** | **Positive(n=8)** | **p** |
| **Age(year)** |  | 63.27±10.34 | 67.79±10.67 | 0.063 | 64.94±12.52 | 65.78±8.7 | 0.81 |  | 64.28±9.6 | 65.69±11.35 | 0.645 | 61.62±11.49 | 62.62±10.92 | 0.847 |
| **AFP (mg/mL)** |  | 2625.72±11381.86 | 23.56±51.01 | 0.235 | 738.17±2182.98 | 1712.32±7032.71 | 0.604 |  | 2074.89±6994.63 | 58.51±166.97 | 0.263 | 777.44±2195.17 | 37.18±53.85 | 0.371 |
| **ALT(IU/L)** |  | 35.45±21.54 | 41.73±37.7 | 0.323 | 49.79±61.36 | 39.63±43.14 | 0.558 |  | 24.35(18.05-34.8) | 25.35(19.85-66.75) | 0.114 | 48.79±61.82 | 32.86±22.39 | 0.506 |
| **AST(IU/L)** |  | 38.46±18.95 | 40.1±25.08 | 0.734 | 67.94±83.7 | 46.86±51.58 | 0.35 |  | 36.89±19.86 | 38.28±18.52 | 0.814 | 58.56±84.47 | 31.78±12.31 | 0.402 |
| **TBIL(µmol/L)** |  | 17.67±19.56 | 16.14±7.8 | 0.692 | 18.42±14.83 | 20.9±13.03 | 0.594 |  | 20.99±24.18 | 20.4±14.66 | 0.928 | 15.32±7.81 | 16.45±6.24 | 0.737 |
| **DBIL(µmol/L)** |  | 6.29±11.68 | 4.94±3.35 | 0.555 | 6.16±6.74 | 6.69±5.79 | 0.798 |  | 7.43±14.52 | 6.14±6.91 | 0.74 | 4.46±2.74 | 4.1±1.59 | 0.747 |
| **Alb (g/L)** |  | 39.63±3.73 | 39.3±3.67 | 0.703 | 38.44±4.06 | 37.72±4.8 | 0.638 |  | 39.44±4.35 | 39.91±3.83 | 0.712 | 38.98±5.09 | 38.76±2.56 | 0.914 |
| **PT (s)** |  | 12.83±1.31 | 12.98±1.06 | 0.59 | 12.83±1.0 | 13.12±1.97 | 0.605 |  | 12.88±1.7 | 12.51±1.01 | 0.422 | 12.93±1.06 | 12.51±0.87 | 0.367 |
| **INR** |  | 1.03±0.1 | 1.04±0.09 | 0.671 | 1.03±0.08 | 1.09±0.19 | 0.333 |  | 1.05±0.16 | 1.04±0.08 | 0.833 | 1.05±0.1 | 1.04±0.06 | 0.693 |
| **Tumor Size(cm)** |  | 4.5(2.5-7.0) | 4.0(2.8-4.9) | 0.025^*^ | 4.58±2.74 | 4.51±2.27 | 0.935 |  | 5.23±2.88 | 4.12±2.07 | 0.176 | 4.73±3.14 | 4.51±1.75 | 0.863 |
| **Sex** |  |  |  | 0.278 |  |  | 0.165 |  |  |  | 0.303 |  |  | 0.317 |
| **Female** |  | 18 | 5 |  | 4 | 2 |  |  | 10 | 2 |  | 5 | 1 |  |
| **Male** |  | 45 | 23 |  | 12 | 21 |  |  | 30 | 14 |  | 11 | 7 |  |
| **HBsAg** |  |  |  | 1 |  |  | 0.515 |  |  |  | 0.462 |  |  | 0.204 |
| **Negative** |  | 18 | 8 |  | 4 | 8 |  |  | 11 | 6 |  | 6 | 1 |  |
| **Positive** |  | 45 | 20 |  | 12 | 15 |  |  | 29 | 10 |  | 10 | 7 |  |
| **Cirrhosis** |  |  |  | 0.55 |  |  | 0.688 |  |  |  | 0.866 |  |  | 0.247 |
| **Absent** |  | 29 | 11 |  | 8 | 13 |  |  | 21 | 8 |  | 10 | 3 |  |
| **Present** |  | 34 | 17 |  | 8 | 10 |  |  | 19 | 8 |  | 6 | 5 |  |
| **Multifocality** |  |  |  | 0.893 |  |  | 0.165 |  |  |  | 0.757 |  |  | 0.526 |
| **Absent** |  | 51 | 23 |  | 12 | 21 |  |  | 31 | 13 |  | 13 | 8 |  |
| **Present** |  | 12 | 5 |  | 4 | 2 |  |  | 9 | 3 |  | 3 | 0 |  |

| **Variables** |  | **KI67(n=145)** | | | | | |  | **P53(n=89)** | | | | | |
| --- | --- | --- | --- | --- | --- | --- | --- | --- | --- | --- | --- | --- | --- | --- |
|  |  | **Training Group(n=101)** | |  | **Test Group(n=44)** | |  |  | **Training Group(n=62)** | |  | **Test Group(n=27)** | |  |
|  |  | **Low(n=48)** | **High(n=53)** | **p** | **Low(n=23)** | **High(n=21)** | **p** |  | **Negative(n=43)** | **Positive(=19)** | **p** | **Negative(n=19)** | **Positive(n=8)** | **p** |
| **Age(year)** |  | 66.58±9.97 | 62.51±10.28 | 0.049^*^ | 66.26±10.44 | 63.86±12.46 | 0.501 |  | 65.33±9.45 | 64.0±8.91 | 0.612 | 64.26±13.78 | 57.62±9.47 | 0.242 |
| **AFP (mg/mL)** |  | 1097.74±5107.24 | 3503.54±12440.81 | 0.22 | 272.73±779.35 | 49.02±55.95 | 0.207 |  | 967.42±5221.72 | 4824.84±17833.01 | 0.207 | 2288.46±7922.54 | 3820.74±9759.11 | 0.684 |
| **ALT(IU/L)** |  | 35.7±24.3 | 35.5±22.61 | 0.965 | 45.63±48.87 | 47.42±56.53 | 0.913 |  | 37.53±35.89 | 37.03±29.48 | 0.958 | 29.7±16.53 | 34.89±15.72 | 0.474 |
| **AST(IU/L)** |  | 39.33±19.14 | 39.97±27.02 | 0.893 | 56.53±52.4 | 56.15±74.55 | 0.985 |  | 41.35±40.33 | 38.44±21.36 | 0.772 | 35.88±16.35 | 40.06±13.76 | 0.547 |
| **TBIL(µmol/L)** |  | 16.13±8.54 | 17.03±11.8 | 0.665 | 18.5±9.01 | 22.67±31.62 | 0.557 |  | 16.05±10.8 | 13.12±5.3 | 0.273 | 14.12±9.89 | 23.74±17.61 | 0.096 |
| **DBIL(µmol/L)** |  | 5.18±4.04 | 5.26±4.67 | 0.929 | 7.1±5.62 | 8.73±19.17 | 0.705 |  | 4.85±3.38 | 3.59±1.62 | 0.133 | 6.42±7.07 | 6.99±8.33 | 0.863 |
| **Alb (g/L)** |  | 38.43±4.44 | 38.66±4.26 | 0.792 | 39.24±3.79 | 38.77±4.94 | 0.726 |  | 38.88±3.79 | 37.73±5.2 | 0.342 | 38.68±4.12 | 41.86±5.1 | 0.114 |
| **PT (s)** |  | 13.04±1.42 | 13.04±1.56 | 0.999 | 12.95±1.05 | 12.77±0.96 | 0.564 |  | 13.11±1.52 | 12.45±1.01 | 0.092 | 13.02±1.44 | 12.8±1.36 | 0.725 |
| **INR** |  | 1.06±0.12 | 1.06±0.14 | 0.901 | 1.04±0.08 | 1.01±0.09 | 0.193 |  | 1.06±0.15 | 1.04±0.09 | 0.632 | 1.02±0.12 | 1.06±0.11 | 0.42 |
| **Tumor Size(cm)** |  | 4.92±2.94 | 4.73±2.61 | 0.73 | 4.78±3.17 | 5.0±2.78 | 0.807 |  | 4.83±2.82 | 4.99±3.18 | 0.844 | 6.22±2.84 | 4.09±2.33 | 0.083 |
| **Sex** |  |  |  | 0.236 |  |  | 0.825 |  |  |  | 0.795 |  |  | 0.43 |
| **Female** |  | 8 | 14 |  | 5 | 4 |  |  | 10 | 5 |  | 5 | 1 |  |
| **Male** |  | 40 | 39 |  | 18 | 17 |  |  | 33 | 14 |  | 14 | 7 |  |
| **HBsAg** |  |  |  | 0.891 |  |  | 0.276 |  |  |  | 0.506 |  |  | 0.551 |
| **Negative** |  | 13 | 15 |  | 9 | 5 |  |  | 15 | 5 |  | 7 | 2 |  |
| **Positive** |  | 35 | 38 |  | 14 | 16 |  |  | 28 | 14 |  | 12 | 6 |  |
| **Cirrhosis** |  |  |  | 0.279 |  |  | 0.49 |  |  |  | 0.122 |  |  | 0.03 |
| **Absent** |  | 26 | 23 |  | 10 | 7 |  |  | 25 | 7 |  | 11 | 1 |  |
| **Present** |  | 22 | 30 |  | 13 | 14 |  |  | 18 | 12 |  | 8 | 7 |  |
| **Multifocality** |  |  |  | 0.359 |  |  | 0.825 |  |  |  | 0.331 |  |  | 0.766 |
| **Absent** |  | 39 | 39 |  | 18 | 17 |  |  | 38 | 15 |  | 13 | 5 |  |
| **Present** |  | 9 | 14 |  | 5 | 4 |  |  | 5 | 4 |  | 6 | 3 |  |

AFP, alpha fetoprotein; ALB, albumin level; ALT, alanine aminotransferase; AST, aspartate aminotransferase; TBIL, total bilirubin; DBIL, directed bilirubin; PT, prothrombin time; INR, international normalized ratio; *, p<0.05.
